# Alcohol-Induced Increases in Inflammatory Cytokines Are Attenuated by Nicotine in Region-Selective Manner in Male Rats

**DOI:** 10.4303/jdar/236036

**Published:** 2017-09-16

**Authors:** Olubukola Kalejaiye, Bruk Getachew, Clifford L. Ferguson, Robert E. Taylor, Yousef Tizabi

**Affiliations:** Department of Pharmacology, College of Medicine, Howard University, Washington, DC 20059, USA

**Keywords:** alcoholism, depression, comorbidity, cytokines, IL-1*β*, TNF-*α*, hippocampus, frontal cortex

## Abstract

**Background:**

Heavy use of alcohol is commonly associated with heavy smoking (nicotine intake). Although many factors, including mood effects of these two drugs may contribute to their co-use, the exact neurobiological underpinnings are far from clear. It is well known that chronic alcohol exposure induces neuroinflammation that may precipitate depressive-like behavior, which is considered an important factor in alcohol relapse. Nicotine, on the other hand, possesses anti-inflammatory and antidepressant effects.

**Purpose:**

In this study, we sought to determine which proinflammatory markers may be associated with the depressogenic effects of chronic alcohol and whether nicotine pretreatment may normalize these changes.

**Study design:**

For this purpose, we treated adult male Wistar rats with alcohol (1.0 g/kg, IP), nicotine (0.3 mg/kg, IP) or their combination once daily for 14 days. Two prominent proinflammatory cytokines (IL-1*β* and TNF-*α*) in two primary brain regions, namely the hippocampus and frontal cortex that are intimately involved in mood regulation, were evaluated.

**Results:**

Chronic alcohol resulted in increases in both cytokines in both regions as determined by Western blot. Nicotine completely blocked alcohol-induced effects in the hippocampus, but not in the frontal cortex. These data suggest that nicotine may mitigate the inflammatory effects of alcohol in brain-selective region. Hence, the previously observed depressogenic effects of alcohol and the antidepressant effects of nicotine may at least be partially mediated through manipulations of proinflammatory cytokines in the hippocampus.

**Conclusion:**

These findings suggest possible therapeutic potential of anti-inflammatory cytokines in combating alcohol-induced depression and/or relapse.

## 1. Introduction

A number of theories have provided plausible explanation for the high coincidence of drinking and smoking. However, the exact neurobiological substrates of such comorbidity is not evident. This might be partially due to the very complex phenomenon of addiction as well as lack of full understanding of the pharmacodynamics of alcohol and nicotine. Nonetheless, it is evident that genetics, various receptor systems (including nicotinic cholinergic) as well as interactions of alcohol and nicotine with the neurotrophic factors, particularly brain-derived neurotrophic factor (BDNF), may play a significant role in their co-use [1,2,3,4]. In addition, some of the opposite pharmacological actions of these two substances, for example, sedative and depressogenic effects of alcohol [1,5], versus nicotine’s stimulant and antidepressant effects [5,6,7] are likely to entice the heavy drinker to smoke in order to counter some of the negative effect associated with high alcohol intake. Indeed, we have provided evidence that the depressogenic effect of alcohol in rats can be blocked by nicotine [2]. Moreover, we and others have reported that the depressogenic effect of alcohol is associated with a reduction in the hippocampal BDNF [1,2,8], which was also reversed by nicotine [2]. However, in addition to the neurotrophic factors, recent findings indicate that modulators of the immune system, particularly the inflammatory mediators, may also play an important role in mood regulation in both animal [4,9] and human studies [10,11]. These findings, not only underscore the complexity of the neurobiological substrates of mood disorders, but may also point to a new therapeutic target.

The current therapeutic interventions in major depressive disorder (MDD), which primarily target the biogenic amines, have known limitations. Thus, the tricyclic antidepressants, monoamine oxidase inhibitors as well as selective serotonin uptake inhibitors, although representing a major advance in treatment of mood disorders, are far from ideal due to their considerable delay in onset of action, unwanted side effects, and significant number of treatment-resistant patients [6,12,13]. Hence, better understanding of the brain mechanism controlling affective behavior in general, and alcohol-induced depression in particular, is warranted.

Although the proinflammatory effects of alcohol have been well documented [14,15,16,17], the exact sites of its action, particularly in relation to its mood altering effects (e.g., induction of depression), are not evident. Similarly, although a number of in vitro and in vivo studies have confirmed anti-inflammatory effects of nicotine, the exact site of its action is yet to be elucidated [18,19,20,21]. Thus, we investigated the effects of these two drugs, alone and in combination, on two prominent proinflammatory cytokines in two primary brain areas. We focused on IL-1*β* and TNF-*α* as these two cytokines have been most extensively studied and have been implicated in various disorders including mood dysregulation [19,22,23]. For brain regions, we chose to study the hippocampus and the frontal cortex as these two areas are of prime importance in the complex circuitry regulating mood [24,25,26]. We hypothesized that chronic intermittent administration of alcohol will be associated with an increase in proinflammatory cytokines and that nicotine will attenuate or block these changes.

## 2. Materials and methods

### 2.1. Animals

Adult male (8–10 week old) Wistar rats were purchased from Harlan Laboratories (now Envigo, Indianapolis, IN, USA) and were housed in groups of two in standard plastic cages with standard bedding in a temperature controlled (24°C–26°C) room. All animals, very close in age, were nonetheless randomized to different ages in all groups.

The animals were kept on a 12-hour reversed light/dark cycle (lights on 7 PM to 7 AM) with free access to food and water. The animals were quarantined for one week (without handling), and then were handled once daily for one week to accustom them to the experimental procedure and to mimic the previous experimental paradigm [2]. A red light source was used for illumination during injection. A total of 24 rats were used. Each experiment consisted of four groups (six rats per group). All experiments were carried out in accordance with NIH guidelines and approved by the Institutional Animal Care and Use Committee.

### 2.2. Drug treatment

As described in detail previously and based on the reported results [1,2,7,27], rats were administered intraperitoneally (IP) ethanol (1 g/kg, 20% v/v), nicotine (0.3 mg/kg base) or their combination daily (between 10 AM–11 AM) for 14 days. The volume of injection was 1 mL/kg for each drug. Ethanol Controls received saline. It is of relevance to note that the chronic daily administration of such alcohol dose results in blood alcohol level (BAL) of approximately 90 mg% (i.e., 90 mg ethanol or alcohol per 100 mL blood, which is higher than the 80 mg% (or 0.08 in legal terms) that is considered “under the influence” in many states) [2].

### 2.3. Tissue preparation

Animals were sacrificed 18 h–20 h after the last injection by decapitation. The brains were rapidly removed, frozen on dry ice and stored at −80 °C. Each frozen brain was later thawed on ice and the frontal cortex and hippocampus were dissected out [2] for western blot analysis.

### 2.4. Western blot

Homogenates of the dissected tissues were made in lysis buffer (10 mM Tris-buffer, 5 mM EDTA, 150 mM NaCl, 0.5% Triton X-100 (v/v) with protease inhibitors (Sigma-Aldrich, St. Louis, MO, USA)). The protein concentration in each sample was determined using a BCA protein Assay Kit (Pierce Biotechnology Inc., IL, USA), and equal protein amount (as confirmed by *β*-actin) was loaded in each immunoblot. The proteins were separated using 12% SDS-PAGE gel and transferred onto a nitrocellulose membrane. The membranes were blocked with a blocking reagent (5% nonfat milk in TBS buffer) for 0.5 h and incubated at 4 °C overnight with the primary antibody against IL-1*β* and TNF-*α* (both at 1:1000 dilution, Santa Cruz Biotechnology Inc., Santa Cruz, CA, USA). The membranes were washed with TBST (TBS buffer with 1% Tween-20) and blocked with the blocking reagent. Membranes were then incubated for 1 h at room temperature in Goat Anti-Rabbit-HRP conjugated secondary antibody (1:300 in TBS, Bio-Rad Laboratories, CA, USA). The membranes were then washed in the TBST washing solution and then visualized using enhanced chemiluminescent kits (Bio-Rad Laboratories, CA, USA). The intensity of the protein bands on the gel was quantified using ChemiDoc XRS system (Bio-Rad Laboratories, CA, USA).

### 2.5. Statistical analysis

Statistical differences between treatment groups were determined by one-way ANOVA followed by post-hoc Newman-Keuls multiple comparison test to determine which groups differed. Significant difference was considered a priori at *P* < .05. Data were analyzed using Graphpad Prism 3 (Graphpad Software, San Diego, CA, USA).

## 3. Results

### 3.1. Effect of nicotine, alcohol, and the combination on hippocampal IL-1*β*

**Figure 1:**
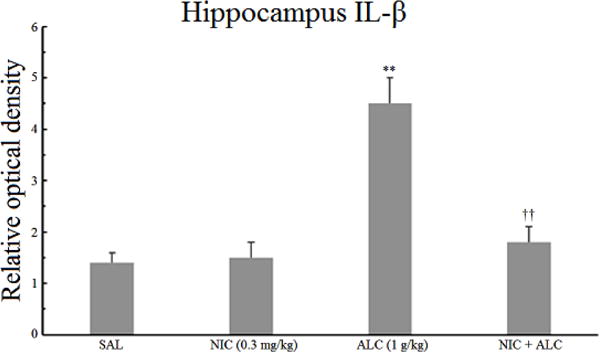
Effect of chronic alcohol, nicotine, and their combination on hippocampal IL-1*β*. Wistar rats were treated daily for 14 days and the samples were obtained 20 h after the last injection. Values are mean ±SEM. **P < .01 compared to controls, ^††^*P* < .01 compared to alcohol only. *N* = 6/group.

Figure 1 depicts the effects of nicotine, alcohol, and the combination on the expression of hippocampal IL-1*β*. A one-way ANOVA indicated a significant main effect *F* (3,24) = 7.62, *P* < .002. Post hoc analysis did not reveal any significant effect of nicotine alone. Alcohol, on the other hand, resulted in a significant increase (3.2 fold) in the relative optical density of IL-1*β* (*P* < .01). Pretreatment with nicotine, however, completely blocked the effect of alcohol on hippocampal IL-1*β* (*P* < .01).

### 3.2. Effect of nicotine, alcohol, and the combination on frontal cortical IL-1*β*

**Figure 2:**
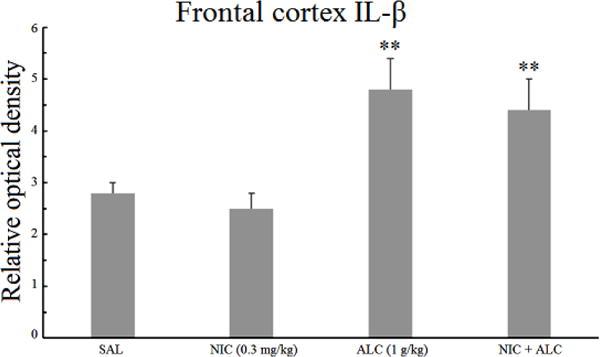
Effect of chronic alcohol, nicotine, and their combination on frontal cortical IL-1*β*. Wistar rats were treated daily for 14 days and the samples were obtained 20 h after the last injection. Values are mean ±SEM. **P < .01 compared to controls, *N* = 6/group.

Figure 2 depicts the effects of nicotine, alcohol, and the combination on the expression of IL-1*β* in the frontal cortex. A one-way ANOVA indicated a significant main effect *F* (3,24) = 5.68, *P* < .005. Post hoc analysis did not reveal any significant effect of nicotine alone. Alcohol, on the other hand, resulted in a significant increase (1.7 fold) in the relative optical density of IL-1*β* (*P* < .01). Pretreatment with nicotine, however, did not alter the effect of alcohol on IL-1*β* expression in the frontal cortex.

### 3.3. Effect of nicotine, alcohol, and the combination on hippocampal TNF-*α*

**Figure 3:**
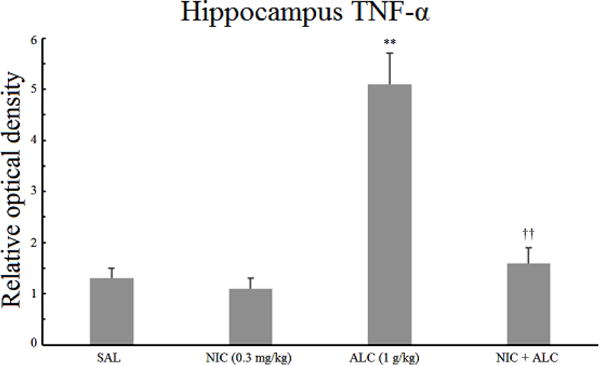
Effect of chronic alcohol, nicotine, and their combination on hippocampal TNF-*α*. Wistar rats were treated daily for 14 days and the samples were obtained 20 h after the last injection. Values are mean ±SEM. **P < .01 compared to controls, ^††^*P* < .01 compared to alcohol only. *N* = 6/group.

Figure 3 depicts the effects of nicotine, alcohol, and the combination on the expression of hippocampal TNF-*α*. A one-way ANOVA indicated a significant main effect *F* (3,24) = 7.85, *P* < .002. Post hoc analysis did not reveal any significant effect of nicotine alone. Alcohol, on the other hand, resulted in a significant increase (3.9 fold) in the relative optical density of TNF-*α* (*P* < .01). Pretreatment with nicotine, however, completely blocked the effect of alcohol on hippocampal TNF-*α* (*P* < .01).

### 3.4. Effect of nicotine, alcohol, and the combination on frontal cortical TNF-*α*

**Figure 4:**
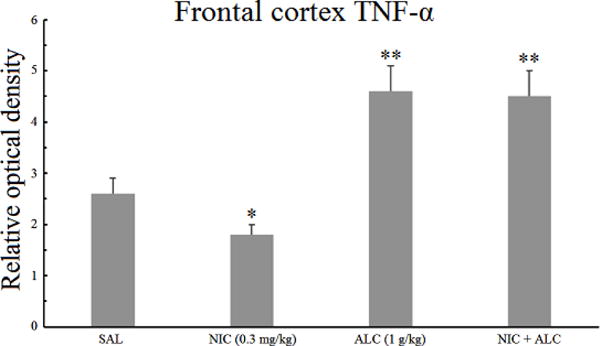
Effect of chronic alcohol, nicotine, and their combination on frontal cortical TNF-*α*. Wistar rats were treated daily for 14 days and the samples were obtained 20 h after the last injection. Values are mean ±SEM. *P < .05, **P < .01 compared to controls. *N* = 6/group.

Figure 4 depicts the effects of nicotine, alcohol, and the combination on the expression of TNF-*α* in the frontal cortex. A one-way ANOVA indicated a significant main effect *F* (3,24) = 5.94, *P* < .005. Post hoc analysis revealed that nicotine alone resulted in 31% decrease (*P* < .05) in frontal cortical TNF-*α*. Alcohol alone resulted in a significant increase (1.7 fold) in the relative optical density of TNF-*α* (*P* < .01). Pretreatment with nicotine, however, did not alter the effect of alcohol on frontal cortical TNF-*α*.

It is of interest to note that the levels of both IL-1*β* and TNF-*α* were 2 fold higher in the frontal cortex compared to the hippocampus (Figures 1–4).

## 4. Discussion

The results of this study indicate that chronic daily treatment of rats with a relatively high dose (BAL = 90 mg%) for two weeks results in an increase in inflammatory markers in both hippocampus and frontal cortex. Thus, the levels of proinflammatory cytokines IL-1*β* and TNF-*α* were elevated by more than 1.7 or 3 fold in the frontal cortex and the hippocampus, respectively, providing further evidence that exposure to chronic intermittent alcohol at above the legal limit can result in a central inflammatory response [14, 15,16,17]. Importantly, however, nicotine pretreatment completely blocked alcohol-induced increases in both cytokines, but only in the hippocampus, suggesting site-specific pharmacodynamic interactions between these two addictive drugs in the brain.

Nicotine per se is very unlikely to be used in therapeutic intervention in alcohol-induced depression as it may actually stimulate the drinking behavior [5]. However, our results suggest that manipulation of specific nicotinic receptors may offer some novel intervention in this regard. Interestingly, varenicline, a partial agonist at alpha4-beta2 nicotinic receptor subtype, may be useful, not only in smoking cessation but also in reducing alcohol intake [28]. However, further work is needed to explore possible nicotinic receptor modulators as therapeutic potential in alcohol-induced depression.

A relevant consequence of such pharmacodynamic interaction between alcohol and nicotine may pertain to their co-abuse. Thus, nicotine’s mitigation of the detrimental effects of alcohol (i.e., inflammatory response and mood dysregulation) could be an additional reason for combining the two together. This study also elucidates a potential brain site where at least some of these interactions may occur. The fact, that nicotine was effective in blocking alcohol effect in the hippocampus only, underscores the significance of this area in mood regulation and highlights the importance of the proinflammatory cytokines in this area. Of significance is also the relative concentrations of various nicotinic receptor subtypes in these two areas. Thus, most centrally abundant nicotinic receptors consisting of alpha4-beta2 and alpha7 subunits are present in both areas [29]. The density of alpha4-beta2 receptor subtype is comparable in both regions [7]. The density of the alpha7 subtype, however, is approximately 30% higher in the hippocampus than the frontal cortex [30]. It remains to be determined whether this differential expression of alpha7 receptor subtype in the hippocampus may be responsible for the observed effect of nicotine in the hippocampus only.

It is also of relevance to note that the concentration of both cytokines in the frontal cortex was twice that of the hippocampus and that nicotine reduced basal TNF-*α* in the frontal cortex. Although the significance of these findings is not evident at this time, it may be postulated that at least some of the diverse effects of nicotine (e.g., cognitive enhancement) might be mediated by its interaction with cortical proinflammatory cytokines [31,32,33,34]. We had previously reported that using the same paradigm, alcohol resulted in depressive-like behavior that was normalized by nicotine pretreatment [2]. Moreover, in the same study, it was observed that the depressogenic effect of alcohol was associated with a reduction in hippocampal BDNF that was also normalized by nicotine.

Thus, it would be of significant interest to explore possible direct interaction between hippocampal proinflammatory cytokines and neurotropic factors, which may not only further our understanding of the neurobiological substrates of drug addiction and/or depression, but may also point to novel intervention. Curiously, it has been postulated that stimulation of inflammatory pathways in general can lead to suppression of neurotropic factors, including BDNF, whereas enhancement of BDNF can suppress the effects of inflammatory cytokines [35,36,37]. Additionally, measurements of the blood cytokine levels could possibly point to a peripheral inflammatory marker of alcohol-induced depression and/or nicotine’s reversal of these effects.

Whether alcohol’s effect is initiated primarily through inflammatory or neurotropic pathways and whether nicotine’s effect follows the same pattern may provide useful hints on common underpinnings of these two addictive and widely used drugs. It is also of importance to note that complex behaviors such as drug addiction and comorbid neuropsychiatric disorders such as depression may involve intricate circuitries that are not only influenced by the inflammatory or neurotrophic factors, but also a number of other neurotransmitter systems [38,39,40,41]. However, whether the final common denominator hinges on the neurotropic and/or inflammatory pathways remains to be elucidated.

In summary, our findings suggest that at least some of the detrimental effects of chronic alcohol may be related to the elevation of proinflammatory cytokines in discrete brain regions and that the counteracting effects of nicotine may be due to its reduction of such cytokines in a brain-selective region such as the hippocampus. These pharmacodynamic interactions may also be a contributing factor to the smoking-drinking comorbidity.

## 5. Conclusions

These findings suggest a role for the hippocampal prionflammatory cytokines in alcohol-induced depression and that targeting such cytokines in this brain region may be of therapeutic potential in alcohol-induced depression and possible prevention of relapse.
